# Polymer-Based Biomaterials for Local Therapy in Cervical Cancer: A Mini-Review

**DOI:** 10.3390/polym18121460

**Published:** 2026-06-11

**Authors:** Mingjing Qiao, Xiaolong Wang, Chenchen Ren, Qian Li, Alaa Hassan, Hakim Boudaoud, Xianhu Liu

**Affiliations:** 1 Tianjian Laboratory of Advanced Biomedical Sciences, National Engineering Research Center for Advanced Polymer Processing Technology, Zhengzhou University, Zhengzhou 450002, China; 2 Équipe de Recherche sur les Processus Innovatifs (ERPI), Université de Lorraine, F-54000 Nancy, France; alaa.hassan@univ-lorraine.fr (A.H.); hakim.boudaoud@univ-lorraine.fr (H.B.)

**Keywords:** cervical cancer, polymer-based biomaterials, local drug delivery, 3D printing, brachytherapy, gynecologic oncology

## Abstract

Cervical cancer continues to require more precise and clinically adaptable local treatment strategies, particularly in the face of insufficient drug accumulation at the lesion site, systemic toxicity of conventional chemotherapy, limited development of postoperative tissue-interfacing platforms, and the anatomical constraints of standard radiotherapy devices. In this mini-review, we summarize the current landscape of polymer-based biomaterials for local therapy in cervical cancer from both materials and clinical perspectives. Specifically, we discuss three interconnected application domains: local drug delivery systems, polymeric scaffolds and tissue-interfacing platforms, and 3D-printed radiotherapy devices. Recent studies indicate that polymer-based local delivery systems, including nanofiber- and hydrogel-based formulations, can improve cervicovaginal retention, controlled release, and local therapeutic exposure. Scaffold-based systems further extend the role of biomaterials by combining sustained local delivery with defect-specific support and tissue interaction, whereas 3D-printed radiotherapy devices contribute primarily through precision enablement, individualized implantation guidance, and improved conformity in anatomically challenging cases. Despite these advances, most available studies remain preclinical or early translational, and important barriers persist in long-term safety, standardization, clinically relevant validation, and workflow integration. Future progress will depend on application-specific design, stronger translational rigor, and closer integration of biomaterials, imaging, and personalized clinical practice.

## 1. Introduction

Cervical cancer remains one of the most important malignancies affecting women worldwide and continues to require effective local treatment across different disease settings [1,2]. According to global cancer statistics, cervical cancer accounted for approximately 660,000 new cases and 350,000 deaths in 2022, with a disproportionate disease burden in low- and middle-income countries [3]. Persistent infection with high-risk human papillomavirus (HPV), especially HPV16 and HPV18, is the principal etiological factor driving cervical carcinogenesis [1]. The disease usually develops through a stepwise process from HPV infection to cervical intraepithelial neoplasia (CIN), including low-grade and high-grade squamous intraepithelial lesions, and eventually invasive cervical cancer if the lesion is not cleared or treated. Histologically, squamous cell carcinoma represents the major subtype, whereas adenocarcinoma and other rare variants account for a smaller but clinically important proportion of cases [4]. As cervical cancer progresses, local invasion, parametrial extension, lymphovascular involvement, and lymph node metastasis become major determinants of treatment strategy and prognosis. Although current management has advanced substantially through surgery [5,6], chemoradiotherapy [7,8], and brachytherapy [9], important limitations remain at the level of local therapeutic intervention. Systemic drug administration (such as paclitaxel) is frequently associated with off-target toxicity and insufficient drug accumulation at the tumor site, whereas currently available local formulations often suffer from leakage, short residence time, poor mucosal retention, and nonuniform distribution within the cervicovaginal region [10]. In addition, postoperative or tissue-interfacing biomaterial strategies for cervical cancer are still at an early stage of development, despite the potential need for local structural support, improved tissue recovery, and sustained therapeutic action in selected settings [11,12]. From the radiotherapy perspective, conventional devices may be inadequate for anatomically complex or individualized cases, highlighting the need for more customized platforms that can improve treatment precision and local conformity [13,14]. Collectively, these challenges support the development of more precise, adaptable, and clinically relevant local therapeutic platforms for cervical cancer.

Polymer-based materials are particularly attractive in this context because their physicochemical and structural properties can be tailored to the demands of local therapy [15,16]. Depending on formulation and design, polymeric materials can enhance local drug retention, enable controlled or sustained release, improve mucoadhesion, provide tissue-interfacing support, and allow patient-specific fabrication [17]. These characteristics make polymers relevant not only as passive carriers, but as multifunctional therapeutic platforms capable of improving spatial precision, local efficacy, and translational flexibility. In cervical cancer, this is especially important because the disease presents a combination of accessible local anatomy and complex therapeutic demands, creating a setting in which biomaterial design may directly influence treatment performance.

**Figure 1:**
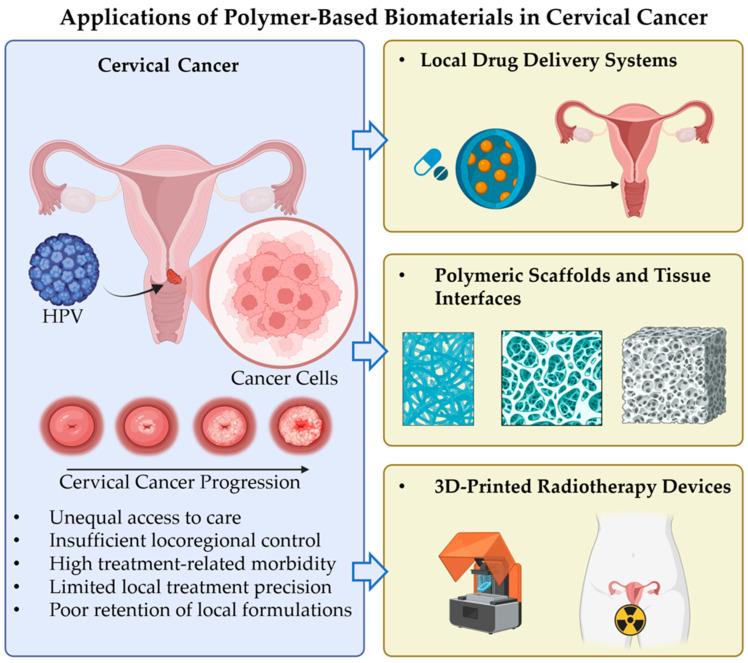
Schematic overview of the major applications of polymer-based biomaterials in cervical cancer. Current polymer-based strategies mainly include local drug delivery systems, polymeric scaffolds and tissue interfaces, and 3D-printed radiotherapy-related devices. Created in BioRender. Lin, Nan. (2026) https://BioRender.com/za71lx0, accessed on 5 June 2026.

As illustrated in Figure 1, the current applications of polymer-based biomaterials in cervical cancer can be broadly organized into three interconnected categories: Local Drug Delivery Systems, Polymeric Scaffolds and Tissue Interfaces, and 3D-Printed Radiotherapy Devices. Local Drug Delivery Systems are being developed to increase drug accumulation at the lesion site while reducing systemic exposure and improving release control [18]. Polymeric Scaffolds and Tissue Interfaces represent an emerging but less developed area, with potential relevance for local support, tissue interaction, healing, and integrated therapeutic functions [19]. 3D-Printed Radiotherapy Devices, including individualized applicators, templates, and related devices, are increasingly important for improving treatment precision and feasibility in anatomically challenging situations [20]. However, to our knowledge, no previous review has provided a dedicated and integrated discussion of polymer-based materials in cervical cancer that simultaneously covers local drug delivery, tissue-interfacing platforms, and 3D-Printed Radiotherapy Devices from both materials and clinical perspectives. Existing reviews have usually addressed these topics separately. For example, previous reviews on vaginal or localized drug delivery for cervical cancer mainly focused on formulations, drug carriers, and pharmacological delivery barriers, but did not substantially discuss cervical scaffold-based tissue interfaces or radiation-enabling devices. Conversely, reviews on 3D printing in gynecological brachytherapy have primarily emphasized applicator fabrication, procedural guidance, and dosimetric improvement, with limited discussion of polymeric drug delivery or postoperative tissue-interfacing platforms. Therefore, the novelty of the present mini-review lies in integrating these three clinically connected but previously separated areas into a single polymer-based biomaterials framework.

In this mini-review, we summarize the current research landscape of polymer-based biomaterials for cervical cancer from both material and clinical perspectives. Rather than focusing on a single formulation type or technical route, we aim to connect biomaterial design with clinically relevant needs, translational challenges, and future development opportunities. By discussing representative advances, unresolved limitations, and possible future directions across three major application areas—local drug delivery systems, polymeric scaffolds and tissue interfaces, and 3D-printed radiotherapy devices—this review seeks to provide a clinically meaningful and forward-looking framework for researchers working at the interface of biomaterials, drug delivery, radiation technology, and gynecologic oncology.

This mini-review was prepared through a narrative literature search in Web of Science using keyword combinations related to cervical cancer, cervicovaginal delivery, polymer-based biomaterials, scaffolds, tissue interfaces, 3D printing, brachytherapy, and radiotherapy applicators. Studies published mainly between 2010 and 2026 were considered, with emphasis on recent literature from 2020 to 2026, and the final selection was refined based on relevance to the three application areas and clinical significance in gynecologic oncology.

## 2. Polymer-Based Biomaterials for Local Therapy in Cervical Cancer

### 2.1. Local Drug Delivery Systems

Local drug delivery is particularly attractive in cervical cancer because the cervix is anatomically accessible through the vaginal route, allowing drugs to be administered close to the lesion [21]. This may help maintain therapeutically relevant local concentrations while reducing systemic exposure and off-target toxicity compared with conventional systemic chemotherapy [22]. Accordingly, localized cervicovaginal delivery has emerged as a rational strategy for improving local therapeutic exposure while limiting systemic toxicity [23]. In local drug delivery, natural polymers such as chitosan, alginate, hyaluronic acid, and cellulose derivatives generally offer good biocompatibility, biodegradability, and mucoadhesion, but may show batch-to-batch variability, weaker control over degradation, and possible biological variability. In contrast, synthetic polymers provide more tunable release kinetics, scalable manufacturing, and reproducible physicochemical properties, although their excipients, degradation products, or long-term mucosal effects require careful safety evaluation.

However, conventional local formulations such as creams, gels, and suppositories are limited by the specific physiological environment of the female reproductive tract [19]. Continuous cervicovaginal mucus turnover, leakage after administration, short residence time, and nonuniform mucosal distribution can all compromise effective drug exposure at the target site. These barriers often necessitate repeated dosing and may still fail to achieve durable local retention. At the same time, reliance on systemic administration to overcome these limitations reintroduces the problem of systemic toxicity, which remains a major concern for cytotoxic agents used in cervical cancer [24]. Against this background, polymer-based local delivery systems have been developed to improve mucoadhesion, prolong retention, and enable controlled or sustained drug release. To address these limitations, a range of polymer-based platforms have been explored, including nanofibers, nanoparticles, in situ gels, liposomes, and hydrogels. In these systems, polymers act not merely as excipients, but as key determinants of release kinetics, vaginal retention, formulation stability, and local safety [19]. The performance of cervicovaginal polymer-based delivery systems is largely governed by a balance between local retention and mucus penetration. In addition to material-related parameters, cervicovaginal delivery performance is also affected by physiological variability of the local environment. Continuous mucus turnover can remove free drugs or drug-loaded carriers from the mucosal surface, thereby shortening residence time, reducing local exposure, and increasing the risk of nonuniform distribution. In reproductive-age patients before ovarian-suppressive treatment, menstrual-cycle-associated changes in mucus volume, hydration, viscosity, pH, and vaginal secretion may further influence formulation retention, particle diffusion, drug release, and mucosal compatibility [25,26,27].

However, in cervical cancer patients, these factors may vary substantially according to age, menopausal status, tumor-related discharge, and prior or ongoing treatments such as surgery, radiotherapy, or chemotherapy. Therefore, polymer-based cervicovaginal delivery systems should ideally be evaluated under physiologically relevant conditions that consider mucus turnover and patient-specific local microenvironmental variability. For prolonged cervicovaginal retention, key physicochemical parameters include mucoadhesive strength, polymer charge, viscosity, swelling behavior, gelation kinetics, mechanical integrity, degradation rate, and the macroscopic geometry of the dosage form. Systems with higher viscosity, stronger mucosal adhesion, appropriate swelling, and slower erosion are generally more favorable for resisting leakage and mucus turnover. In contrast, efficient mucus penetration is mainly determined by nanoscale features, including particle size, surface charge, hydrophilicity, surface shielding, and colloidal stability. Small, non-aggregating, hydrophilic particles with near-neutral surface charge, particularly mucus-inert surfaces, are less likely to bind strongly to mucins and therefore can diffuse more effectively through the mucus mesh [28,29]. These two requirements are not always aligned: strong mucoadhesion may improve residence time but may also hinder deep mucus transport, whereas mucus-inert nanoparticles may penetrate mucus but require a retained depot or scaffold to avoid rapid clearance. Therefore, rational cervicovaginal delivery design often requires a hierarchical strategy, such as combining a mucoadhesive or structurally retained polymer matrix with mucus-penetrating nanoscale drug carriers.

Among recent representative studies, Duan et al. developed a composite intravaginal delivery system in which paclitaxel nanocrystals were first prepared, subsequently coated with polydopamine, further modified with polyethylene glycol (PEG), and finally incorporated into electrospun nanofibers [30]. This multistep design was intended to combine the advantages of nanocrystal-based drug loading, PEG-assisted transmucosal transport, and nanofiber-mediated local retention. Such a strategy directly addresses one of the major challenges in cervicovaginal drug delivery, namely the need to achieve both sufficient mucus penetration and prolonged residence at the local site. In a murine cervicovaginal tumor model, the PEGylated nanocrystal-loaded nanofibers exhibited longer vaginal residence, improved transmucosal penetration, minimal mucosal irritation, and stronger tumor inhibition than the corresponding formulation without PEG modification. Mechanistically, this study is particularly noteworthy because it integrates nanocrystal engineering with a polymeric fibrous depot, thereby providing a balanced approach to local persistence and tissue penetration in cervical cancer therapy.

**Figure 2:**
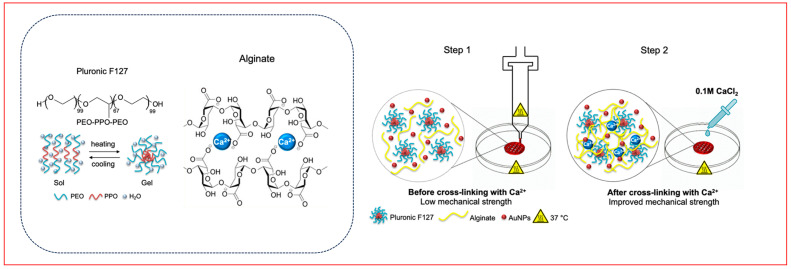
Schematic illustration of a thermosensitive Pluronic F127–alginate hydrogel system for vaginal delivery of therapeutic nanoparticles, showing the sol–gel transition, calcium-mediated cross-linking, and potential compatibility with 3D printing for structurally adaptable local administration [31]. Reprinted with permission from Ref. [31]. Copyright 2026, American Chemical Society.

A second representative example was reported by Kiseleva et al., who developed a thermosensitive Pluronic F127–alginate hydrogel for vaginal delivery of therapeutic nanoparticles to cervical cancer (Figure 2) [31]. As illustrated in the figure, the system combines the thermoresponsive sol–gel transition behavior of Pluronic F127 with the ionic cross-linking capability of alginate, and the final formulation can be further stabilized by calcium-mediated cross-linking. This design moves beyond conventional semisolid formulations by introducing not only local drug delivery capability but also structural conformity and potential adaptability for individualized application. Notably, the authors further demonstrated that the hydrogel was compatible with 3D printing, with high geometric precision and minimal deviation from the intended design. From the perspective of polymer-based biomaterials, this study is important because it links drug delivery with structural customization, suggesting that hydrogel-based platforms may serve not only as local carriers for sustained release but also as adaptable systems for improved formulation placement and local anatomical fitting.

Taken together, these two studies illustrate two distinct but complementary design directions in polymer-based local drug delivery for cervical cancer. Electrospun nanofiber-based platforms are particularly attractive for achieving prolonged local retention while incorporating engineered nanodrugs with enhanced mucus-penetrating properties. In contrast, Figure 2 highlights the potential of hydrogel-based systems to integrate local delivery with conformability, cross-linking-enhanced mechanical properties, and even printable structural customization. Collectively, these advances indicate that polymer-based local delivery systems are evolving from simple topical formulations toward multifunctional platforms that combine sustained release, mucoadhesion or retention, tissue conformity, and translational flexibility.

Critically, most current polymer-based local delivery systems for cervical cancer remain at the preclinical stage, and their superiority is often demonstrated under simplified experimental conditions. Important translational gaps include limited validation in dynamic cervicovaginal mucus, insufficient evaluation of tumor microenvironment-associated barriers, unclear effects on the vaginal microbiota, and the lack of standardized models for residence time, mucosal safety, and clinically meaningful therapeutic efficacy. Therefore, future studies should move beyond retention and release profiles alone and assess whether these systems provide durable therapeutic benefit under physiologically relevant cervicovaginal and tumor conditions.

### 2.2. Polymeric Scaffolds and Tissue Interfaces

The potential value of polymeric scaffolds and tissue-interfacing platforms arises from clinical situations in which local therapy alone may be insufficient, and additional structural support, defect coverage, tissue integration, or prolonged local therapeutic action may be desirable. This is particularly relevant after cervical conization or other tissue-removing procedures, where biomaterial-based interfaces may help bridge local defects while simultaneously serving as carriers for sustained therapeutic delivery [32]. For cervical tissue-interfacing scaffolds, natural polymers or decellularized matrices may better support cell interaction, extracellular matrix remodeling, and biological integration, but they often have limited mechanical tunability and greater variability. Synthetic polymers provide more controllable stiffness, degradation rate, architecture, and manufacturing reproducibility, but may lack intrinsic bioactivity and may require surface modification to reduce inflammatory or fibrotic responses.

From a biomaterial’s perspective, scaffolds and tissue interfaces differ from conventional local formulations in that their function is not limited to drug release [33,34]. Instead, these systems are intended to provide physical contact with the injured or treated site, support cell attachment or tissue coverage, and in some cases combine structural repair with local therapeutic action [35,36]. For cervical applications, this concept is attractive because the local anatomy is accessible, the defect geometry may be patient-specific, and the desired platform may need to balance biocompatibility, mechanical compliance, degradability, and manufacturability. For cervical tissue-interfacing scaffolds, mechanical matching should be understood as functional rather than exact modulus matching. Because cervical tissue mechanics vary with anatomical region, age, parity, hormonal status, disease state, and prior treatment, no universal stiffness threshold has been established. Ideally, implanted scaffolds should be compliant enough to conform to the cervical surface or postoperative defect and avoid compression, irritation, or stress concentration, while maintaining sufficient structural integrity for defect coverage, local retention, and controlled release during the intended residence period. Mechanical mismatch may also influence the host response and subsequent cervical remodeling. Excessively stiff, slowly degradable, or highly cross-linked scaffolds may aggravate foreign body reactions, macrophage-driven inflammation, fibrotic encapsulation, excessive collagen deposition, or scar-like remodeling [37,38]. Conversely, overly soft or rapidly eroding scaffolds may collapse or fail to provide durable tissue contact. Therefore, future cervical scaffold design should consider not only initial tensile, compressive, or viscoelastic properties, but also degradation-dependent mechanical evolution, inflammatory response, collagen organization, epithelial regeneration, and the risk of fibrosis or cervical stenosis.

However, compared with the more established literature on local vaginal formulations, cervical scaffold-based strategies remain relatively sparse, and most available studies are still proof-of-concept or preclinical.

**Figure 3:**
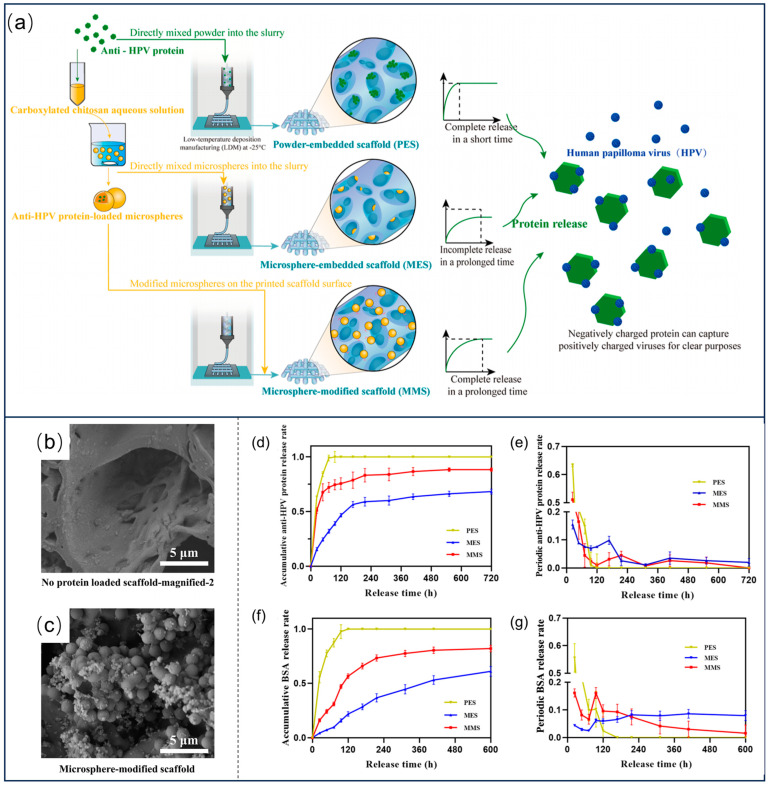
Representative polymer-based cervical scaffold design for tissue-interfacing local therapy after cervical conization. (**a**) Schematic illustration of three anti-HPV protein loading strategies in a patient-customized 3D-printed cervical scaffold, including powder-embedded scaffold (PES), microsphere-embedded scaffold (MES), and microsphere-modified scaffold (MMS), together with their distinct release profiles. (**b**,**c**) Scanning electron microscopy images showing the porous microstructure of the unloaded scaffold and the surface morphology after microsphere modification. (**d**,**e**) Cumulative and periodic release profiles of the anti-HPV protein from PES, MES, and MMS. (**f**,**g**) Cumulative and periodic release profiles of bovine serum albumin (BSA) as a model protein from the corresponding scaffold systems [12]. Reprinted with permission from Ref. [12]. Copyright 2026, American Chemical Society.

Among recent representative studies, Ji et al. reported a patient-customized 3D-printed cervical implant scaffold based on carboxylated chitosan for sustained anti-HPV protein delivery after cervical conization (Figure 3a–g) [12]. In this work, the scaffold functioned not merely as a passive implant, but as a tissue-interfacing platform designed for postoperative defect coverage and local therapeutic release. As shown in Figure 3a, the authors compared three loading modes—powder-embedded scaffold (PES), microsphere-embedded scaffold (MES), and microsphere-modified scaffold (MMS)—thereby demonstrating that the mode of incorporation substantially influenced release behavior. Structural characterization confirmed successful scaffold modification (Figure 3b,c). As shown in Figure 3d–g, direct powder incorporation resulted in rapid and nearly complete release, whereas microsphere incorporation slowed release but remained incomplete over time. In contrast, the microsphere-modified scaffold (MMS) achieved a more favorable profile, combining prolonged release with a relatively complete cumulative release behavior, both for the anti-HPV protein and for the model protein BSA. From the perspective of biomaterial design, this is a notable finding because it indicates that surface modification with drug-loaded microspheres may provide a more balanced strategy for sustained postoperative delivery than simple direct embedding. This study is conceptually important because it illustrates how a polymer-based cervical scaffold can integrate local structural repair and sustained site-specific therapy, thereby extending the role of biomaterials beyond conventional topical administration.

A biologically informative study by Li et al. investigated partial reconstruction of the uterine cervix in a rat model using a decellularized human cervical scaffold combined with adipose-derived stem cells [39]. Although this work was based on a biologic extracellular matrix scaffold rather than a defined synthetic polymeric system, it remains relevant to the present discussion because it illustrates the regenerative rationale for tissue-interfacing cervical scaffolds.

Overall, these studies suggest that polymeric scaffolds and tissue-interfacing platforms may occupy an important intermediate space between local drug delivery and structural intervention. On one hand, they can serve as localized depots for sustained therapeutic release; on the other, they may provide defect-specific support and encourage local tissue integration. At present, however, this direction remains much less mature than local drug delivery systems or 3D-printed radiotherapy devices. Key unresolved issues include limited disease-specific evidence in cervical oncology, insufficient long-term in vivo validation, uncertain degradation-performance matching in the cervical environment, and the lack of clinically standardized fabrication pathways. Future progress in this area will likely depend on more disease-oriented scaffold design, better integration of therapeutic and regenerative functions, and stronger validation in clinically relevant postoperative models.

From a critical perspective, cervical scaffold research is still much less mature than conventional tissue engineering applications in skin, bone, or cartilage. The clinical indication for scaffold implantation after cervical procedures remains insufficiently defined, and key issues such as mechanical matching, degradation-dependent support, inflammatory response, fibrosis risk, epithelial regeneration, and cervical remodeling have not been systematically evaluated. As a result, future scaffold studies should be driven by specific cervical clinical scenarios rather than generic scaffold design alone.

### 2.3. 3D-Printed Radiotherapy Devices

Radiotherapy remains a cornerstone of local treatment in cervical cancer, and brachytherapy is particularly important for achieving adequate dose escalation while limiting irradiation of surrounding pelvic organs [9,40]. However, standard applicators and templates are not always sufficient in anatomically complex or clinically challenging cases. Irregular vaginal anatomy, asymmetric residual disease, parametrial extension, or recurrent pelvic tumors may all reduce the fit of conventional devices and compromise needle placement, target coverage, or organ-at-risk sparing [41]. In this context, 3D-printed radiotherapy devices have emerged as a clinically relevant extension of polymer-based biomaterials, because they allow patient-specific geometry, rapid customization, and potentially improved conformity between the device and the individual anatomy [13,14].

From a functional perspective, these devices differ from local drug delivery systems and tissue-interfacing scaffolds in that their primary role is not to release therapeutic agents or promote tissue regeneration, but to enable more precise radiation delivery. In gynecologic brachytherapy, 3D printing has mainly been used to fabricate individualized applicators, interstitial needle templates, vaginal molds, and related accessories that help standardize insertion geometry and optimize dosimetry. Systematic and narrative reviews published in 2023 concluded that gynecologic tumors, especially cervical cancer, represent the most common clinical setting for 3D printing in brachytherapy, and that the major reported benefits include improved applicator fit, better target coverage, and more flexible treatment planning in difficult cases [34].

**Figure 4:**
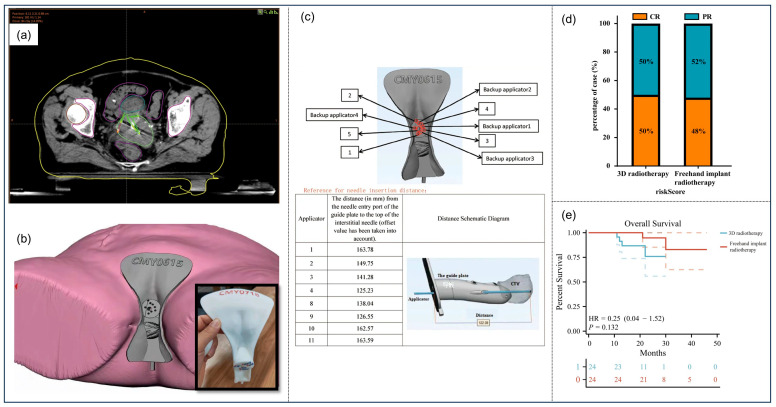
Representative workflow and preliminary clinical outcomes of patient-specific 3D-printed radiotherapy devices for cervical cancer brachytherapy [42]. (**a**) CT-based target delineation and treatment planning for individualized 3D-printed IC/IS brachytherapy. (**b**) Three-dimensional reconstruction and placement of the customized vaginal applicator/guide device, with an inset photograph of the printed device. (**c**) Applicator report showing patient-specific device information, predefined needle channels, and reference insertion distances used for implantation guidance and procedural standardization. (**d**) Comparison of short-term therapeutic response between the 3D radiotherapy group and the freehand implant radiotherapy group, expressed as complete response (CR) and partial response (PR). (**e**) Kaplan–Meier analysis of overall survival in the two treatment groups. Together, these data illustrate the translational pathway of 3D-printed radiotherapy devices from imaging-based preplanning and individualized fabrication to guided implantation and preliminary clinical outcome evaluation. Reproduced from Lu et al. [42] under the terms of the Creative Commons Attribution-NonCommercial-NoDerivatives 4.0 International License.

Among recent representative studies, Lu et al. investigated the clinical application of patient-specific 3D-printed brachytherapy guide devices in cervical cancer and provided a useful example of how polymer-based radiotherapy devices can be translated into clinical practice (Figure 4a–e) [42]. As illustrated in Figure 4a,b, the workflow integrates pre-treatment imaging and three-dimensional reconstruction with the fabrication of an individualized applicator or guide plate that conforms to patient-specific pelvic anatomy. The printed device is then used to assist applicator placement and needle insertion in anatomically complex cases, thereby improving procedural reproducibility and supporting more precise target-oriented implantation. The degree of customization is further reflected in the applicator report shown in Figure 4c, which includes individualized device information and predefined needle insertion distances, highlighting the practical value of 3D printing for procedure planning and standardization. From a clinical perspective, the importance of this work lies less in claiming that 3D printing is universally superior to conventional freehand implantation, and more in demonstrating that customized radiotherapy devices can be feasibly incorporated into cervical brachytherapy workflows. As summarized in Figure 4d,e, the study further compared treatment responses and survival outcomes between the 3D radiotherapy group and the freehand implant radiotherapy group, providing preliminary clinical evidence for the safety and effectiveness of the personalized approach. For the present mini-review, this study is particularly relevant because it illustrates the full translational pathway of polymer-based radiotherapy devices—from image-guided design and device fabrication to individualized implantation and clinical outcome evaluation. Conceptually, it reinforces the view that the main value of 3D-printed radiotherapy devices in cervical cancer lies in precision enablement, namely, improving anatomical fit, procedural guidance, and treatment standardization in selected patients for whom conventional devices may be suboptimal.

A related and increasingly important adjunctive direction is the use of polymeric gel or hydrogel spacers to improve organ separation during cervical brachytherapy. Although spacers are not always 3D-printed, they belong to the broader category of polymer-based radiation-enabling devices because their function is to physically increase the distance between the cervix or target volume and nearby organs such as the rectum. A 2024 phase I clinical trial reported that hydrogel application effectively increased the cervix-to-rectum distance and reduced rectal dose during brachytherapy for cervical cancer without short-term severe adverse events [43]. This line of work is conceptually important because it shows that polymer-based devices in radiotherapy are not limited to applicator fabrication alone; they may also include temporary space-modifying materials that improve the therapeutic ratio.

For radiation-compatible printing applications, material selection should prioritize low atomic number, near-water-equivalent attenuation, CT/MRI compatibility, biocompatibility, sterilization tolerance, dimensional stability, and smooth surface quality. Medical-grade photopolymer resins for SLA/DLP printing and polyamide-based materials for SLS printing are commonly attractive for patient-specific applicators or templates, whereas PLA and ABS may be useful for prototyping or selected validated devices but require careful dosimetric, biocompatibility, and sterilization assessment before clinical use. In terms of resolution, microscopic precision is usually unnecessary; instead, submillimeter-to-millimeter geometric accuracy is required to preserve anatomical fit, needle-channel position, source path reproducibility, and dosimetric reliability. Therefore, each printed device should undergo imaging-based geometric verification and, when needed, dosimetric quality assurance before clinical application [44,45].

Taken together, current 3D-printed radiotherapy devices in cervical cancer mainly represent a strategy of precision enablement rather than direct antitumor material action. Their value lies in helping clinicians adapt radiotherapy hardware to patient-specific anatomy, improve applicator or template performance, and in some cases reduce dose to adjacent organs through spacer-assisted separation. At present, however, the evidence base remains limited by relatively small case series, heterogeneous device designs, variable printing workflows, and the absence of broadly standardized manufacturing and quality-assurance pathways. Future progress will likely depend on stronger prospective validation, closer integration with image-guided adaptive brachytherapy, and clearer clinical criteria for selecting patients most likely to benefit from customized or spacer-assisted approaches.

Although 3D-printed radiotherapy devices have clearer clinical feasibility than many drug-delivery or scaffold systems, their current evidence base remains limited by small cohorts, heterogeneous device designs, institution-specific workflows, and variable quality-assurance procedures. Importantly, these devices primarily improve precision, anatomical fit, and procedural reproducibility rather than exerting direct antitumor material activity. Future studies should therefore define which patient subgroups truly benefit from customized devices and should validate material compatibility, geometric accuracy, dosimetric reliability, sterilization, and regulatory compliance in prospective clinical workflows.

## 3. Conclusions and Future Perspectives

Although polymer-based biomaterials have already shown considerable promise in cervical cancer, the field is still far from maturity. At present, the major challenge is no longer simply to demonstrate that polymers can be used for local delivery, tissue interfacing, or device fabrication, but to determine which platform is most suitable for which clinical scenario, and under what conditions these systems can provide meaningful advantages over existing practice. In this sense, future progress will depend less on increasing material complexity alone and more on improving clinical relevance, translational robustness, and application-specific design.

For local drug delivery systems, the next stage of development should move beyond proof-of-concept retention or release studies toward platforms that are better matched to the real cervicovaginal environment. This includes improving the reproducibility of local residence, clarifying how mucus turnover and anatomical variability influence performance, and designing systems that can maintain efficacy without sacrificing patient comfort or usability. More importantly, future studies should focus on whether these formulations can provide durable therapeutic benefit in clinically relevant settings, rather than only demonstrating favorable release kinetics or short-term antitumor activity. Another promising but still underexplored direction is the development of stimuli-responsive polymeric delivery systems capable of releasing drugs in response to lesion-associated cues such as acidic pH, reactive oxygen species, enzymatic activity, or hypoxia. However, their application in cervical cancer remains insufficiently established, and potential safety issues, including mucosal irritation, polymer- or excipient-related toxicity, and disruption of the vaginal microbiota, should be carefully evaluated before clinical translation [46,47].

For polymeric scaffolds and tissue-interfacing platforms, the key opportunity lies in developing systems that do more than fill a structural gap. In cervical applications, the most promising direction may be the creation of multifunctional postoperative interfaces that combine defect-specific support, controlled local delivery, and biologically favorable tissue interaction. However, this area also requires greater conceptual precision: future scaffold design should be driven by clearly defined clinical needs, such as post-conization repair, anti-HPV maintenance therapy, or local healing support, rather than by generic scaffold construction alone. Long-term validation in realistic postoperative models will be especially important if these systems are to move beyond exploratory studies.

For 3D-printed radiotherapy devices, the future is likely to depend on how effectively personalization can be integrated into routine brachytherapy workflows. Customized applicators, templates, and related devices are clinically appealing because they address anatomical complexity directly, but their broader adoption will require more standardized preplanning pipelines, faster manufacturing, clearer quality-assurance procedures, and stronger evidence regarding which patients truly benefit from individualized devices. In parallel, polymer-based spacer technologies may expand the role of biomaterials in radiotherapy by improving organ separation and therapeutic ratio, especially when incorporated into image-guided and adaptive treatment strategies.

From a regulatory perspective, implantable polymer systems and 3D-printed radiotherapy-related devices must also satisfy device-specific requirements for biocompatibility, sterility, degradation products, leachables, mechanical reliability, dimensional reproducibility, and manufacturing quality control. Future translational studies should therefore consider early alignment with regulatory frameworks, such as FDA guidance for additive-manufactured medical devices, ISO 10993-based biological evaluation [48], and quality management requirements, rather than treating material performance and regulatory validation as separate steps.

Looking ahead, one of the most important trends will be the convergence of biomaterials, imaging, and personalization. Rather than remaining separate categories, local drug depots, tissue-interfacing constructs, and radiotherapy-assisting devices may increasingly evolve into hybrid platforms with combined functions. Such integration could be particularly valuable in cervical cancer, where treatment often involves anatomically accessible lesions, multimodal management, and the need to balance efficacy with preservation of surrounding tissues. Achieving this goal, however, will require much closer collaboration between clinicians, materials scientists, engineers, and manufacturers than is currently seen in most proof-of-concept studies. Overall, the future of polymer-based biomaterials in cervical cancer should not be defined simply by “more materials” or “more complex systems”, but by better alignment between biomaterial design and real clinical problems. The most impactful advances will likely come from platforms that are simpler, more reproducible, easier to standardize, and more clearly linked to specific therapeutic needs. If this translational direction can be achieved, polymer-based biomaterials may become an increasingly important part of precision local therapy for cervical cancer.

To make future development more actionable, polymer-based strategies for cervical cancer can be considered along different translational timelines. In the near term, mucoadhesive hydrogels, nanofiber depots, and nanoparticle-loaded local systems appear most realistic for local drug delivery, provided that they can demonstrate reproducible cervicovaginal residence, controlled release over clinically meaningful periods, mucosal safety, and minimal disturbance of the vaginal microbiota. In the medium term, cervical tissue-interfacing scaffolds may be useful for selected postoperative settings, but their translation will require defined mechanical targets, degradation-dependent support, fibrosis assessment, and validation in large-animal cervicovaginal models rather than rodents alone. In contrast, highly complex stimuli-responsive or cell-loaded scaffolds may face lower translational probability if their triggers are insufficiently disease-specific, their degradation or immune response is difficult to control, or their manufacturing and regulatory pathways become overly complex. Among the three directions, 3D-printed radiotherapy-related devices currently have the clearest near-term clinical pathway, because their primary function is geometric guidance and precision enablement rather than direct biological modulation. Future studies should therefore include phase I trials focused on safety, device fit, mucosal tolerance, and procedural feasibility, followed by phase II studies evaluating local control, residence or placement reproducibility, dosimetric benefit, patient comfort, and treatment-related toxicity.

## Data Availability

No new data were created or analyzed in this study. Data sharing is not applicable to this article.
